# Nomogram for predicting papillary thyroid carcinoma risk combined with clinical, biochemical and ultrasonographic indicators

**DOI:** 10.1186/s12885-026-15686-z

**Published:** 2026-02-05

**Authors:** Hui Li, Pengwei Lou, Yuting Huang, Jing Ma, Tingting Zhang, Li Ma

**Affiliations:** 1 Department of Endocrinology, The Fourth Clinical Medical College of Xinjiang Medical University, Urumqi, 830000 China; 2 Department of Big Data, College of Information Engineering, Xinjiang Institute of Engineering, Urumqi, 830023 China; 3 Department of Medical Administration, The Fourth Clinical Medical College of Xinjiang Medical University, Urumqi, 830000 China

**Keywords:** Papillary thyroid carcinoma (PTC), Risk factors, Prediction model, Nomogram, Thyroid nodules

## Abstract

**Background:**

Epidemiological studies have shown an increasing trend in the incidence of papillary thyroid cancer (PTC). Therefore, the question of how to make clinical decisions when an investigation reveals atypical or suspicious cells arises. The aim of this study was to develop a nomogram for assessment of the individual risk of malignancy of thyroid nodules based on clinical, biochemical and ultrasonographic indicators.

**Methods:**

This retrospective study analyzed data from 2993 patients who had undergone thyroid surgery, with patients randomly allocated to a training cohort (*n* = 2095) and a validation cohort (*n* = 898) at a ratio of 7:3. Predictor selection was conducted through a two-step process: first, univariate analysis identified variables significantly associated with PTC (*P* < 0.05). Subsequently, these significant variables were entered into a multivariate logistic regression analysis to determine independent risk factors. The final nomogram prediction model was constructed based on the coefficients of these independent factors. The model’s performance was rigorously evaluated via internal validation. Discrimination was assessed by the area under the receiver operating characteristic curve (AUC), calibration was evaluated using calibration curves accompanied by the Hosmer-Lemeshow goodness-of-fit test, and clinical utility was examined through decision curve analysis (DCA).

**Results:**

Univariate analysis selected twenty-two variables that may be factors affecting the occurrence of PTC (*P* < 0.05). Multivariate analysis determined younger age (odd ratio [OR] = 0.956, 95% confidence interval [CI], 0.945–1.574, *P* < 0.001), drinking (OR = 1.862, 95%CI, 1.013–3.469, *P* = 0.047), enlarged cervical lymph nodes (ECLN) (OR = 1.586, 95%CI, 1.116–2.283, *P* = 0.012), hypoechoic (OR = 29.07, 95%CI, 14.572–66.880, *P* < 0.001), irregular shape (OR = 6.838, 95%CI, 4.945–9.534, *P* < 0.001), calcification (OR = 1.583, 95%CI, 1.124–2.233, *P* = 0.008), higher thyroid-stimulating hormone (TSH)(OR = 1.141, 95%CI, 1.053–1.237, *P* = 0.001) and lower serum potassium (K) (OR = 0.478, 95%CI, 0.327–0.695, *P* < 0.001) were finalized as risk factors for PTC (*P* < 0.05). A nomogram of PTC was constructed based on influencing factors. The nomogram underwent internal validation, which showed good discrimination in the training and validation groups, with an area under the curve (AUC) of 0.869 (95% CI, 0.853–0.886) and 0.872 (95% CI, 0.847–0.897), respectively. The calibration of the prediction model was evaluated by the Hosmer-Lemeshow goodness-of-fit test; the Hosmer-Lemeshow goodness-of-fit test values were *P* = 0.923 and *P* = 0.608, respectively. This indicates that the model aligns well with the observed data. The clinical utility assessed by decision curve analysis (DCA) demonstrates that the nomogram provides a superior net benefit across a wide range of threshold probabilities compared to alternative strategies. This indicates that the nomogram is a clinically useful tool for selecting patients who would benefit from intervention.

**Conclusion:**

A nomogram capable of accurately quantifying the risk of papillary thyroid carcinoma in thyroid nodules was developed by integrating clinical, biochemical, and ultrasonographic indicators. This predictive tool is designed to assist clinicians in optimizing clinical decision-making and avoiding unnecessary invasive procedures.

## Introduction

Thyroid cancer is the most common endocrine malignant tumor, with a globally increasing incidence over the past three decades [1, 2]. Papillary thyroid carcinoma (PTC) represents the most prevalent histological subtype [3, 4]. Notably, projections indicate a continuing rise in the age-standardized incidence rate (ASIR) for thyroid cancer in both China and the global population in the coming years [5]. This increasing trend, partly attributable to enhanced diagnostic scrutiny [6], has resulted in a substantial clinical burden in managing thyroid nodules and cancer, particularly in China [7]. Despite the high incidence of thyroid malignancies, most thyroid nodules with suspected malignancies are eventually pathologically-proven to be benign. Therefore, it is crucial to identify malignant and benign thyroid nodules to avoid unnecessary overtreatment such as fine needle aspiration (FNA) biopsy and surgery [8, 9]. The preoperative management, assessment of risk factors and ultrasound examination have always been the focus of scholars in the preoperative research field of papillary thyroid cancer.

To date, there is no reliable method of accurately quantifying the risk of malignancy in specific patient populations. American Thyroid Association guidelines and Thyroid Imaging Reporting and Data System (TI-RADS) are considered as the main criteria for determining malignancy and are generally followed by radiologists in practice [10, 11]. However, these classification systems were established based on fine-needle thyroid puncture (FNA) cytology results, which mainly included data from nodules of >1 cm. In addition, there are a few reports indicating that inflammatory factors, metabolic indicators, serum thyroid stimulating hormone (TSH) or positive thyroid autoantibodies may be a predictor of thyroid malignancy [12, 13]. However, these guidelines or related studies rely on relatively single indicators. They either used FNA cytology results for their final diagnoses, which are less reliable than those confirmed via surgical inspection, or they include a relatively small number of patients. Most of these studies have focused on one or several specific risk factors, such as clinical, biochemical, ultrasound, or radiological factors. Only a very few studies have comprehensively analyzed these risk factors.

Experienced clinicians can predict the risk of malignancy using demographic, clinical, biochemical, ultrasound, and cytological characteristics. Individualized risk quantification would be helpful for both clinical decision making and counseling patients. The aim of this study was, therefore, to create a predictive nomogram based on clinical, biochemical, and ultrasonographic indicators to accurately predict the probability of malignancy.

## Materials and methods

### Study design and participants

The clinical data of 4171 patients with thyroid nodules, as confirmed by surgical pathology, admitted to The Hospital of Traditional Chinese Medicine Affiliated to Xinjiang Medical University between January 2018 and December 2021, were retrospectively retrieved. Inclusion criteria: (1) The patient had undergone thyroid surgery; (2) Complete clinical data, biochemical data and thyroid ultrasound imaging data were available; (3) After histopathological confirmation of the nature of all the nodules, according to the pathological type, patients with papillary thyroid carcinoma and benign thyroid nodules were included. The exclusion criteria included: (1) Patients aged below 18 years (pediatric population); (2) Pregnant or lactating women; (3) Patients with incomplete clinical data; (4) Patients with abnormal data deemed as outliers; (5) Patients who had undergone a previous thyroid operation; (6) Patients with a concomitant diagnosis of other malignant tumors; (7) Patients with subacute thyroiditis or Hashimoto’s thyroiditis.

**Fig. 1:**
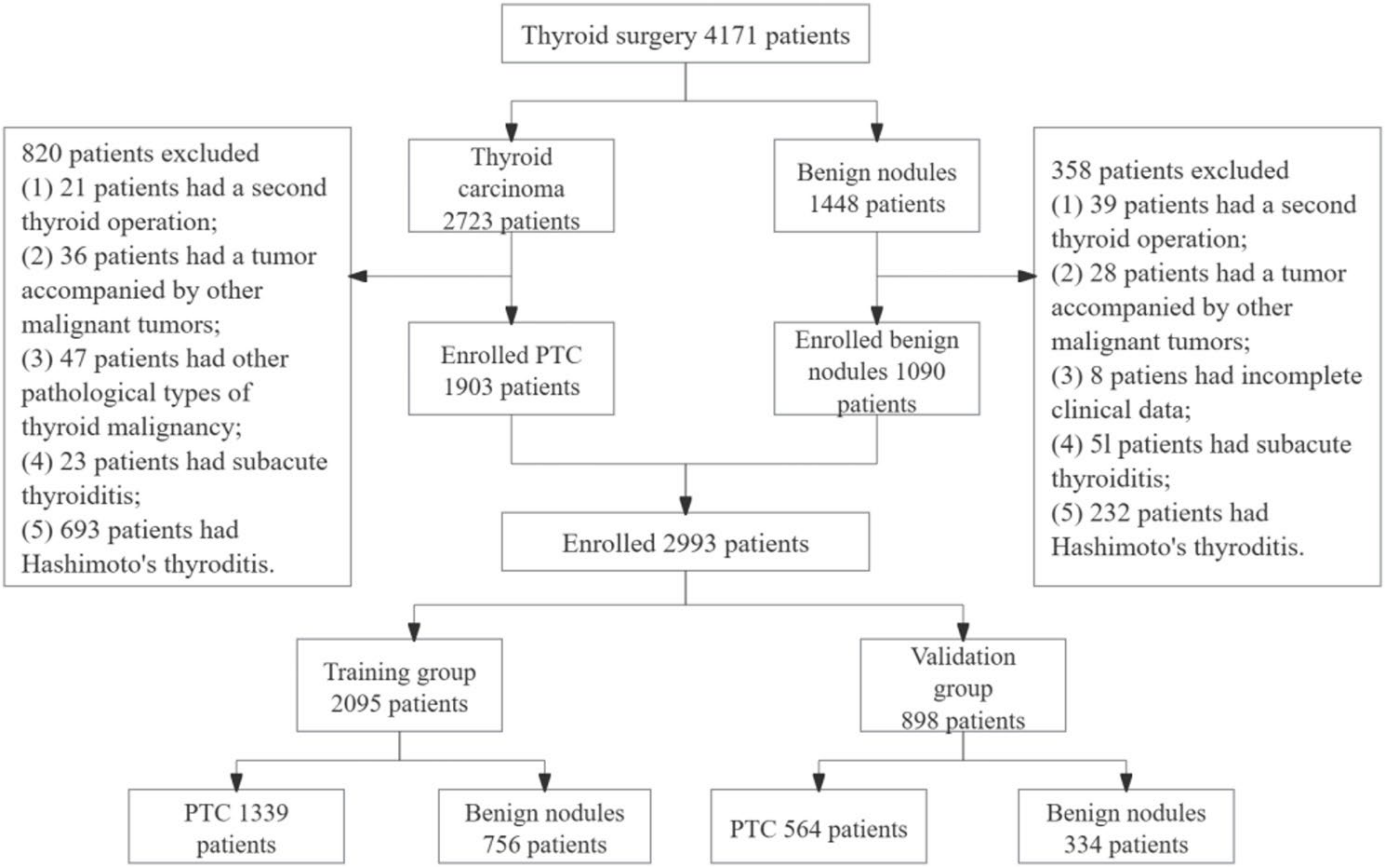
Flow diagram of the data screening

After strict data filtering and processing, 2993 samples were obtained and distributed into two groups (training group: 2095; validation group: 898) according to a ratio of 7:3. The detailed results are shown in Fig. 1. The histopathological diagnosis of papillary thyroid carcinoma was utilized as the outcome variable in this study. A nomogram was constructed based on the training group and was verified using the validation group. The study was approved by the Medical Ethics Committee of The Hospital of Traditional Chinese Medicine Affiliated to Xinjiang Medical University (Ethical approval number: 2022XE0164). Given the retrospective design of this study, formal informed consent was waived for the use of existing research data in accordance with the institutional review board guidelines. However, all the patients subsequently undergoing surgery signed the informed consent forms for these procedures. The study was performed in accordance with the Declaration of Helsinki.

### Information collection

The general clinical conditions recorded included: age, sex, body mass index (BMI), ethnicity, marital status, hypertension, diabetes, heart disease, smoking, drinking, family history of cancer, family history of thyroid disease, time since the first incidence of a thyroid nodule. Biochemical variables were recorded in routine blood tests: peripheral inflammation in peripheral blood (platelet to lymphocyte ratio (PLR), neutrophil to lymphocyte ratio (NLR), lymphocyte to monocyte ratio (LMR)), triglyceride(TG), total cholesterol (CHOL), high density lipoprotein cholesterol (HDL), low density lipoprotein cholesterol (LDL), blood urea nitrogen (BUN), serum creatinine (Scr), uric acid (UA), fasting blood glucose (FPG), electrolyte (K, Na, Ca, Mg, Cl, P), thyroid function (total thyroxine (TT4), total triiodothyronine (TT3), free thyroxine (FT4), free triiodothyronine (FT3), (TSH), thyrotrophin receptor antibody (TRAb), thyroglobulin (Tg), calcitonin (CT), tumor markers (alpha fetoprotein) (AFP), carcinoembryonic antigen (CEA), CA50, CA125, CA725, CA153, CA199, Cyfra21.1, SCC, NSE). The clinical details of all of the above patients were retrieved from the hospital database.

A GE-E11 ultrasound system (GE Medical System, USA) with a linear array probe was used to acquire ultrasound images in the frequency range of 9-11 MHz, and the images were recorded on PACS workstations. All patients were in the supine position, with the lower shoulder neck neck extended to better expose the inferior thyroid rim. Scans of both thyroid lobes and the isthmus were obtained in both transverse and longitudinal planes. Longitudinal and transverse images of the thyroid were obtained according to American College of Radiology accreditation standards. The thyroid nodule ultrasound chart was prejudged by two experienced sonographers. The following USG parameters of the nodules were recorded: (1) Margin: Categorized as ‘clear’ (well-defined smooth border) or ‘unclear’ (ill-defined or irregular border including spiculation/microlobulation); (2) Shape: Classified as ‘regular’ (oval/round) or ‘irregular’ (taller-than-wide appearance, defined as anteroposterior diameter exceeding transverse diameter in the transverse plane); (3) Echogenicity: Classified according to ACR TI-RADS criteria and further grouped into two categories for analysis: ‘Anechoic/Hyperechoic or isoechoic’ versus ‘Hypoechoic/Very hypoechoic’ (relative to thyroid parenchyma). No very hypoechoic nodules were identified in our study cohort. (4) Intranodal hyperechogenicity: Documented as present (bright echogenic foci without acoustic shadowing) or absent; (5) Calcification: Recorded as present (any bright echogenic foci with or without acoustic shadowing) or absent; (6) Intranodular central flow: Assessed via color Doppler and classified as present (vascularity within the nodule interior) or absent; (7) Nodular position; (8) Enlarged cervical lymph nodes (ECLN): Documented as present or absent.

Postoperative histopathologic evaluations were performed by pathologists experienced in thyroid pathology. The histopathologic results of the patients operated on were grouped as PTC or benign (other pathological types of thyroid malignancies were not included in this study).

### Statistical analysis

Statistical analyses were performed with the open-source R software Version 4.1.3 (https://www.r-project.org). Pearson’s chi-square or Fisher’s exact tests, and the Student’s t-test were used for univariate analysis. Categorical variables were expressed as patient numbers and percentages (%), and continuous variables were expressed with mean standard deviation (SD). Multivariate logistic regression analysis was used to assess the independent risk factors for PTC. Odds ratios (ORs) with 95% confidence intervals (CI) were presented for factors that were statistically significant in univariate analysis. The indicators screened by multivariate analysis were plotted as a nomogram. The discriminative ability of our nomogram was assessed using Harrell’s Concordance Index (C-index), which has a similar meaning to the area under the curve (AUC), and the threshold range is 0.5-1; the larger C-index means perfect discrimination. Calibration was performed to compare how well the predicted probabilities from our nomogram matched the actual probabilities of PTC. Quantification of the net benefit under different thresholds and analysis of the decision curve enabled the determination of the nomogram’s clinical practicability. All tests were two-sided, and p-values less than 0.05 were considered statistically significant.

## Results

### Baseline characteristics

In this study, a total of 2095 patients with postoperative thyroid nodules were included in the training group according to the inclusion and exclusion criteria. There were 561 males and 1534 females; the average age was (49.15 ± 12.01) years of age. Postoperative pathology confirmed 1339 patients with PTC and 756 patients with benign nodules. In the validation group, a total of 898 patients were included in the analysis, of which there were 235 males and 663 females The average age was (49.41 ± 11.49) years of age. Postoperative pathology confirmed 564 patients with PTC and 334 patients with benign nodules. There were no statistically significant differences in general clinical data, biochemical indexes and ultrasound characteristics between the training set and the validation set except for (pls check) FT4, monocyte (MONO), Scr and Mg. (Table 1).

**Table 1:** Clinical characteristics of the study population (*N* = 2993)

Characteristics	Total *n* = 2993	Training group *n* = 2095	Validation group *n* = 898	$\:{x}^{2}$or t-test	*P* value
Gender				0.119	0.730
Male	796 (26.60)	561 (26.78)	235 (26.17)		
Female	2197 (73.40)	1534 (73.22)	663 (73.83)		
Age (years)	49.23 ± 11.85	49.15 ± 12.01	49.41 ± 11.49	0.544	0.587
BMI (kg/m^2^)	25.16 ± 3.63	25.22 ± 3.62	25.03 ± 3.64	0.730	0.181
Ethnicity				0.548	0.760
Han	2249 (75.14)	1572 (75.04)	677 (75.39)		
Uygur	392 (13.10)	280 (13.36)	112 (12.47)		
Other nationalities	352 (11.76)	243 (11.60)	109 (12.14)		
Hypertension				0.268	0.605
Yes	752 (25.12)	532 (25.39)	220 (24.50)		
No	2241(74.88)	1563 (74.61)	678 (75.50)		
Diabetes				0.847	0.357
Yes	326 (10.89)	221 (10.55)	105 (11.69)		
No	2667 (89.11)	1874 (89.45)	793 (88.31)		
Heart disease				2.214	0.137
Yes	244 (8.15)	181 (8.64)	63 (7.02)		
No	2749 (91.85)	1914 (91.36)	835 (92.98)		
Smoking				0.709	0.400
Yes	404 (13.50)	290 (13.84)	114 (12.69)		
No	2589 (86.50)	1805 (86.16)	784 (87.31)		
Drinking				0.001	0.971
Yes	339 (11.33)	237 (11.31)	102 (11.36)		
No	2654 (88.67)	1858 (88.69)	796 (88.64)		
Family history of cancer				0.002	0.962
Yes	418 (13.97)	293 (13.99)	125 (13.92)		
No	2575 (86.03)	1802 (86.01)	773 (86.08)		
Family history of thyroid disease				0.626	0.429
Yes	70 (2.34)	46 (2.20)	24 (2.67)		
No	2923 (97.66)	2049 (97.80)	874 (97.33)		
Location				3.211	0.360
Upper pole	540 (18.04)	374 (17.85)	166 (18.48)		
Middle third	1283 (42.87)	897 (42.81)	386 (42.98)		
Lower pole	1049 (35.05)	747 (35.66)	302 (33.63)		
Isthmus	121 (4.04)	77 (3.68)	44 (4.91)		
Enlarged cervical lymph nodes (ECLN)				0.030	0.862
Yes	492 (16.44)	346 (16.52)	752 (83.74)		
No	2501 (83.56)	1749 (83.48)	146 (16.26)		
Boundary				0.156	0.693
Clear	1463 (48.88)	1029 (49.12)	434 (48.33)		
Unclear	1530 (51.12)	1066 (50.88)	464 (51.67)		
Shape				0.051	0.821
Regular	1119 (37.39)	786 (37.52)	333 (37.08)		
Irregular	1874 (62.61)	1309 (62.48)	565 (62.91)		
Echogenicity				0.509	0.476
Hypoechoic/Very hypoechoic	2573 (85.97)	1810 (86.40)	763 (84.97)		
Anechoic/Hyperechoic or isoechoic	416 (14.03)	285 (13.60)	131 (15.03)		
Intranodular Hyperechoic				0.262	0.609
Yes	1271 (42.47)	896 (42.77)	375 (41.76)		
No	1722 (57.53)	1199 (57.23)	523 (58.24)		
Calcification				0.178	0.673
Yes	886 (29.60)	625 (29.83)	261 (29.06)		
No	2107 (71.40)	1470 (70.17)	637 (70.94)		
Central intranodular flow				0.117	0.732
Yes	1126 (53.75)	784 (37.42)	342 (38.08)		
No	1867 (46.25)	1311 (62.58)	556 (61.92)		
Time since the first incidence of a thyroid nodule	0.88 ± 3.63	0.88 ± 2.08	0.89 ± 1.99	0.157	0.875
TT3 (nmol/L)	1.68 ± 0.36	1.68 ± 0.35	1.67 ± 0.40	-0.656	0.512
TT4 (nmol/L)	93.43 ± 17.49	93.69 ± 17.24	92.82 ± 18.04	-1.254	0.210
FT3 (pmol/L)	4.71 ± 0.91	4.72 ± 0.89	4.69 ± 0.95	-0.515	0.606
FT4 (pmol/L)	15.81 ± 2.57	15.88 ± 2.62	15.64 ± 2.46	-2.322	0.020
TSH (uIU/ml)	2.37 ± 1.58	2.37 ± 1.60	2.35 ± 1.56	-0.669	0.504
TR-Ab (Iu/L)	0.68 ± 1.54	0.67 ± 1.35	0.71 ± 1.91	0.593	0.553
CA50 (U/ML)	8.25 ± 7.69	8.34 ± 7.97	8.02 ± 6.98	-1.060	0.289
AFP (ng/ml)	3.46 ± 3.08	3.43 ± 3.16	3.52 ± 2.88	0.680	0.497
CEA (ng/ml)	1.62 ± 1.20	1.63 ± 1.24	1.60 ± 1.12	-0.435	0.663
CA125 (U/ml)	12.94 ± 14.45	12.83 ± 14.08	13.20 ± 15.30	0.647	0.518
CA153 (U/ml)	9.52 ± 5.06	9.49 ± 4.94	9.59 ± 5.34	0.542	0.588
CA199 (U/ml)	11.02 ± 10.10	11.19 ± 10.69	10.60 ± 8.55	-1.477	0.140
CA724 (U/ml)	4.39 ± 10.84	4.50 ± 11.95	4.14 ± 7.62	-0.818	0.414
Cyfra21.1 (ng/ml)	2.05 ± 1.15	2.04 ± 1.14	2.06 ± 1.18	0.283	0.777
NSE (ng/ml)	15.45 ± 5.67	15.45 ± 5.83	15.48 ± 5.29	0.147	0.883
SCC (ng/ml)	0.83 ± 0.62	0.83 ± 0.76	0.83 ± 0.54	0.399	0.690
CT (pg/ml)	1.24 ± 1.45	1.24 ± 1.47	1.23 ± 1.40	-0.291	0.771
TG (mmol/L)	1.00 ± 1.11	1.01 ± 1.15	0.98 ± 0.99	-0.481	0.630
CHOL (mmol/L)	3.86 ± 0.97	3.86 ± 0.98	3.85 ± 0.94	-0.255	0.799
HDL (mmol/L)	0.86 ± 0.39	0.86 ± 0.38	0.84 ± 0.41	-1.377	0.169
LDL (mmol/L)	1.99 ± 0.78	1.98 ± 0.78	2.03 ± 0.78	1.804	0.071
WBC (10^9/L)	6.31 ± 1.74	6.29 ± 1.72	6.37 ± 1.78	1.141	0.254
RBC (10^12/L)	4.70 ± 0.50	4.69 ± 0.50	4.71 ± 0.49	1.036	0.300
HGB (g/L)	138.47 ± 16.54	138.48 ± 16.64	138.45 ± 16.31	-0.052	0.958
HCT (L/L)	0.42 ± 0.04	0.42 ± 0.04	0.42 ± 0.04	0.179	0.858
MCV (fL)	89.14 ± 5.52	89.23 ± 5.42	88.95 ± 5.74	-1.266	0.205
MCH (pg)	29.53 ± 2.38	29.57 ± 2.35	29.45 ± 2.46	-1.331	0.183
MCHC (g/L)	330.94 ± 13.12	331.04 ± 13.26	330.71 ± 12.78	-0.617	0.537
PLT	256.31 ± 64.06	255.84 ± 63.97	257.40 ± 64.28	0.611	0.541
LYMP (10^9/L)	1.96 ± 0.59	1.96 ± 0.60	1.97 ± 0.59	0.309	0.757
MONO (10^9/L)	0.41 ± 1.56	0.40 ± 0.15	0.42 ± 0.16	2.431	0.015
NEUT (10^9/L)	3.78 ± 1.38	3.76 ± 1.38	3.81 ± 1.39	0.909	0.363
RDW-CV (%)	12.98 ± 1.25	12.97 ± 1.24	13.01 ± 1.26	0.738	0.461
RDW-SD (fL)	42.09 ± 3.13	42.09 ± 3.08	42.07 ± 3.26	-0.168	0.866
PLR	140.47 ± 51.02	140.48 ± 51.47	140.45 ± 49.99	-0.018	0.986
NLR	2.07 ± 0.98	2.06 ± 0.99	2.07 ± 0.97	0.123	0.902
LMR	5.94 ± 12.14	6.12 ± 13.33	5.52 ± 8.77	-1.236	0.217
BUN (mmol/L)	4.51 ± 1.41	4.50 ± 1.44	4.53 ± 1.33	0.514	0.608
Scr (umol/L)	67.56 ± 14.03	67.11 ± 14.46	68.62 ± 12.93	2.699	0.007
UA (umol/L)	304.68 ± 85.52	305.80 ± 85.21	303.76 ± 85.59	-0.434	0.664
K (mmol/L)	3.38 ± 0.49	3.38 ± 0.49	3.39 ± 0.49	0.338	0.735
Na (mmol/L)	13.59 ± 0.49	13.59 ± 0.49	13.58 ± 0.49	-0.781	0.435
CI (mmol/L)	10.67 ± 7.35	10.77 ± 7.89	10.44 ± 5.90	-0.123	0.218
Ca (mmol/L)	1.99 ± 0.08	2.00 ± 0.07	1.99 ± 0.100	-1.417	0.157
Mg (mmol/L)	0.04 ± 0.20	0.05 ± 0.21	0.03 ± 0.17	-2.266	0.024
P (mmol/L)	0.87 ± 0.34	0.87 ± 0.34	0.88 ± 0.33	4.467	0.640

Note: *BMI* Body Mass Index, *ECLN* Enlarged Cervical Lymph Nodes, *TSH* Thyroid-Stimulating Hormone, *FT3* Free Triiodothyronine, *FT4* Free Thyroxine, *TT3* Total Triiodothyronine, *TT4* Total Thyroxine, *PLR* Platelet-to-Lymphocyte Ratio, *NLR* Neutrophil-to-Lymphocyte Ratio, *LMR* Lymphocyte-to-Monocyte Ratio, *TG* Triglycerides, *CHOL* Total Cholesterol, *HDL* High-Density Lipoprotein, *LDL* Low-Density Lipoprotein, *BUN* Blood Urea Nitrogen, *Scr* Serum Creatinine, *UA* Uric Acid, *FPG* Fasting Plasma Glucose

### Statistical descriptions and univariate analysis

The descriptions and univariate analysis of factors, including general clinical features, such as gender and age, and biochemical and ultrasonographic factors, are listed in Table 2. In the training cases, univariate analysis showed that twenty-three factors differed significantly between the participants with PTC and those with benign nodules: age, BMI, heart disease, smoking, drinking, nodular location, ECLN, margin, shape, echogenicity, intranodular hyperechogenicity, calcification, intranodular central flow, the first incidence of the discovery of a thyroid nodular, TT3, TSH, HDL, RBC, MCV, MCH, RDW-SD, UA and K (all *P* < 0.05). Meanwhile, the results of single variable analysis in the validation group are shown in Table 2 for comparison.

**Table 2:** Univariate analysis of the relationship between PTC and benign nodules

	Training group		$\:{x}^{2}$or t-tes	*P* value	Validation group		$\:{x}^{2}$or t-tes	*P* value
	PTC *n* = 1339	Benign nodules *n* = 756			PTC *n* = 564	Benign nodules *n* = 334		
Gender			3.590	0.058			2.183	0.140
Male	377	184			157	78		
Female	962	572			407	256		
Age	47.59 ± 11.26	51.92 ± 12.77	7.785	< 0.001	47.76 ± 10.91	52.19 ± 11.92	5.679	< 0.001
BMI	25.40 ± 3.64	24.90 ± 3.56	-3.027	0.003	25.15 ± 3.67	24.83 ± 3.64	-1.270	0.205
Ethnicity			0.819	0.664			1.367	0.505
Han	1011	561			429	248		
Uygur	179	101			72	40		
Other nationalities	149	94			63	46		
Hypertension			0.695	0.404			2.488	0.115
Yes	348	184			148	72		
No	991	572			416	262		
Diabetes			0.111	0.739			0.430	0.512
Yes	139	82			69	36		
No	1200	674			495	298		
Heart disease			4.910	0.027			0.024	0.878
Yes	102	79			39	24		
No	1237	677			525	310		
Smoking			5.405	0.020			0.833	0.361
Yes	203	87			76	38		
No	1136	669			488	296		
Drinking			14.512	< 0.001			4.370	0.037
Yes	179	59			73	28		
No	1161	697			491	306		
Family history of cancer			1.312	0.252			0.089	0.766
Yes	196	97			80	45		
No	1143	659			484	289		
Family history of thyroid disease			0.651	0.420			0.680	0.410
Yes	32	14			17	7		
No	1307	742			547	327		
Location			36.884	< 0.001			19.68	< 0.001
Upper pole	286	88			128	38		
Middle third	525	372			244	162		
Lower pole	478	269			182	120		
Isthmus	50	27			30	14		
Enlarged cervical lymph nodes (ECLN)			40.36	< 0.001			14.43	< 0.001
Yes	273	73			112	34		
No	1066	683			452	300		
Margin			381.63	< 0.001			163.61	< 0.001
Clear	443	586			180	254		
Unclear	896	170			384	80		
Shape			669.428	< 0.001			294.93	< 0.001
Regular	227	559			89	244		
Irregular	1112	197			475	90		
Echogenicity			534.071	< 0.001			252.734	< 0.001
Hypoechoic/Very hypoechoic	1331	479			563	204		
Anechoic/Hyperechoic or isoechoic	8	277			1	130		
Intranodular Hyperechogenicity			139.24	< 0.001			51.939	< 0.001
Yes	701	195			287	88		
No	636	561			277	246		
Calcification			97.95	< 0.001			49.07	< 0.001
Yes	499	126			210	51		
No	840	630			354	283		
Intranodular central flow			57.89	< 0.001			27.98	< 0.001
Yes	582	554			252	90		
No	757	202			312	244		
Time since the first incidence of a thyroid nodule	0.66 ± 1.55	1.25 ± 2.73	5.435	< 0.001	0.63 ± 1.54	1.34 ± 2.52	5.221	< 0.001
TT3	1.66 ± 0.35	1.70 ± 0.34	2.249	0.031	1.64 ± 0.31	1.72 ± 0.52	3.178	0.002
TT4	93.43 ± 16.78	94.17 ± 18.04	0.923	0.356	91.82 ± 16.48	94.51 ± 20.32	2.162	0.031
FT3	4.72 ± 0.99	4.70 ± 0.69	-0.542	0.588	4.64 ± 0.62	4.75 ± 1.32	1.792	0.070
FT4	15.89 ± 2.70	15.87 ± 2.47	-0.177	0.859	15.62 ± 2.33	15.69 ± 2.68	0.459	0.646
TSH	2.46 ± 1.57	2.26 ± 1.65	-2.728	0.006	2.42 ± 1.48	2.22 ± 1.60	-1.828	0.068
TR-Ab	0.64 ± 0.68	0.72 ± 2.06	1.167	0.243	0.63 ± 0.47	0.83 ± 3.06	1.168	0.244
CA50	8.03 ± 7.63	8.61 ± 8.53	1.913	0.051	8.19 ± 7.49	7.73 ± 6.03	-0.969	0.333
AFP	3.44 ± 3.52	3.43 ± 2.39	-0.052	0.959	3.47 ± 2.09	3.61 ± 3.88	0.690	0.490
CEA	1.59 ± 1.03	1.68 ± 1.53	1.208	0.207	1.58 ± 1.09	1.64 ± 1.17	0.815	0.415
CA125	12.89 ± 14.12	12.72 ± 14.01	-0.254	0.799	12.76 ± 10.50	13.95 ± 21.05	1.128	0.259
CA153	9.52 ± 4.85	9.44 ± 5.09	0.777	0.723	9.48 ± 5.11	9.79 ± 5.68	0.865	0.387
CA199	10.98 ± 9.65	11.71 ± 12.28	1.854	0.060	10.74 ± 8.07	10.36 ± 7.81	-0.620	0.535
CA724	4.59 ± 13.96	3.92 ± 7.13	-1.610	0.214	3.98 ± 5.72	4.43 ± 10.08	0.849	0.396
Cyfra21.1	2.01 ± 1.10	2.10 ± 1.21	1.709	0.086	2.03 ± 1.21	2.10 ± 1.12	0.879	0.379
NSE	15.36 ± 5.11	15.59 ± 6.93	0.794	0.427	15.36 ± 5.61	15.67 ± 4.71	0.859	0.390
SCC	0.83 ± 0.53	0.83 ± 0.56	0.012	0.990	0.86 ± 0.92	0.80 ± 0.35	-1.075	0.283
CT	1.25 ± 1.45	1.24 ± 1.49	-0.119	0.905	1.25 ± 1.45	1.20 ± 1.32	-0.479	0.632
TG	1.55 ± 1.06	1.48 ± 1.15	-1.396	0.163	1.51 ± 0.94	1.50 ± 0.96	-0.189	0.850
TC	4.33 ± 0.92	4.41 ± 0.94	1.781	0.075	4.34 ± 0.86	4.37 ± 0.92	0.604	0.546
HDL	1.26 ± 0.28	1.29 ± 0.28	2.700	0.007	1.24 ± 0.28	1.27 ± 0.30	1.681	0.093
LDL	2.47 ± 0.72	2.48 ± 0.74	0.438	0.661	2.53 ± 0.69	2.51 ± 0.72	-0.400	0.689
WBC	6.31 ± 1.70	6.25 ± 1.76	-0.758	0.449	6.42 ± 1.80	6.28 ± 1.76	-1.099	0.272
RBC	4.71 ± 0.52	4.65 ± 0.47	-2.521	0.012	4.74 ± 0.51	4.66 ± 0.45	-2.124	0.028
HGB	138.75 ± 17.33	138.01 ± 15.33	-1.013	0.311	138.74 ± 17.03	137.96 ± 15.03	-0.716	0.474
HCT	0.42 ± 0.04	0.42 ± 0.04	-1.000	0.317	0.42 ± 0.04	0.42 ± 0.04	-0.948	0.343
MCV	88.99 ± 5.45	89.63 ± 5.34	2.573	0.010	88.67 ± 5.94	89.43 ± 5.36	1.920	0.054
MCH	29.49 ± 2.39	29.71 ± 2.26	1.998	0.046	29.34 ± 2.50	29.62 ± 2.38	1.652	0.099
MCHC	331.06 ± 13.38	330.99 ± 13.06	-0.131	0.896	330.59 ± 12.78	330.93 ± 12.79	0.388	0.698
PLT	256.06 ± 64.69	255.44 ± 62.73	-0.213	0.831	257.47 ± 65.10	257.29 ± 62.97	-0.039	0.969
LYMPH	1.96 ± 0.61	1.96 ± 0.59	0.065	0.948	1.98 ± 0.58	1.93 ± 0.61	-1.358	0.175
MONO	0.40 ± 0.15	0.40 ± 0.16	-0.424	0.671	0.42 ± 0.16	0.41 ± 0.17	-1.077	0.282
NEUT	3.78 ± 1.37	3.72 ± 1.39	-1.086	0.278	3.84 ± 1.41	3.77 ± 1.34	-0.754	0.451
RDW-CV	12.97 ± 1.22	12.98 ± 1.27	0.201	0.841	13.00 ± 1.32	13.01 ± 1.16	-0.103	0.918
RDW-SD	41.97 ± 3.01	42.32 ± 3.18	2.557	0.011	41.91 ± 3.35	42.36 ± 3.08	2.029	0.043
PLR	140.68 ± 51.90	140.13 ± 50.73	-0.236	0.814	139.51 ± 51.33	142.87 ± 47.62	1.118	0.264
NLR	2.08 ± 0.99	2.03 ± 0.99	-1.029	0.304	2.06 ± 0.97	2.09 ± 0.96	0.549	0.583
LMR	5.72 ± 9.43	6.86 ± 18.32	1.596	0.111	5.71 ± 10.94	5.21 ± 2.08	-0.280	0.412
BUN	4.92 ± 1.36	5.04 ± 1.46	1.861	0.063	4.94 ± 1.27	5.03 ± 1.34	0.945	0.338
Scr	70.49 ± 15.68	69.83 ± 25.45	-0.736	0.462	70.16 ± 12.67	69.10 ± 11.46	-1.282	0.200
UA	305.80 ± 85.21	293.14 ± 74.72	-3.526	< 0.001	303.76 ± 85.59	294.97 ± 74.83	-1.610	0.120
K	3.89 ± 0.32	3.95 ± 0.33	3.867	< 0.001	3.89 ± 0.31	3.94 ± 0.32	1.925	0.055
Na	140.48 ± 2.24	140.56 ± 2.17	0.715	0.475	140.36 ± 2.08	140.41 ± 2.08	0.312	0.755
CI	106.29 ± 2.30	106.44 ± 2.35	1.441	0.150	106.51 ± 2.22	106.37 ± 2.26	-0.857	0.392
Ca	2.28 ± 0.12	2.29 ± 0.13	1.673	0.095	2.27 ± 0.12	2.29 ± 0.11	1.660	0.097
Mg	0.87 ± 0.08	0.87 ± 0.09	1.937	0.053	0.87 ± 0.07	0.86 ± 0.07	0.471	0.293
P	1.19 ± 0.18	1.18 ± 0.18	-0.269	0.788	1.19 ± 0.17	1.17 ± 0.18	-1.482	0.139

Note: *BMI* Body Mass Index, *ECLN* Enlarged Cervical Lymph Nodes, *TSH* Thyroid-Stimulating Hormone, *FT3* Free Triiodothyronine, *FT4* Free Thyroxine, *TT3* Total Triiodothyronine, *TT4* Total Thyroxine, *PLR* Platelet-to-Lymphocyte Ratio, *NLR* Neutrophil-to-Lymphocyte Ratio, *LMR* Lymphocyte-to-Monocyte Ratio, *TG* Triglycerides, *CHOL* Total Cholesterol, *HDL* High-Density Lipoprotein, *LDL* Low-Density Lipoprotein, *BUN* Blood Urea Nitrogen, *Scr* Serum Creatinine, *UA* Uric Acid, *FPG* Fasting Plasma Glucose

### Training and validation of a PTC nomogram

**Fig. 2:**
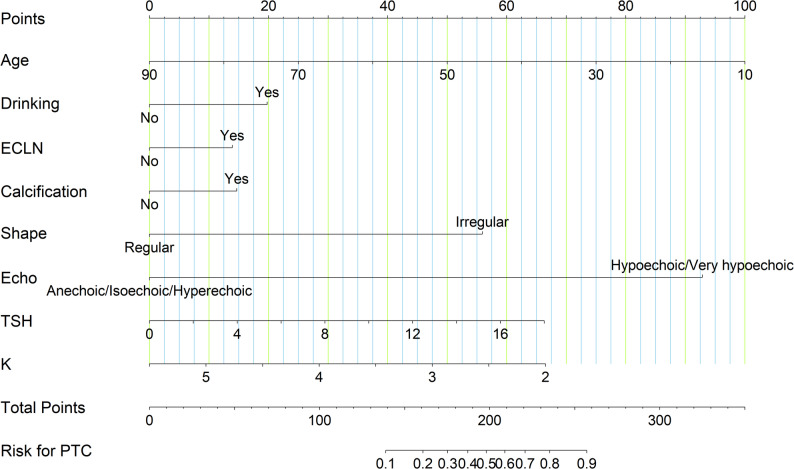
Nomogram to predict the risk of PTC. Notes: To use the nomogram, the patient’s value for each variable on the corresponding axis is located, and a line is drawn upward to determine the number of points received for each variable value. The sum of these numbers is located on the total points axis to determine the risk of PTC

The above twenty-two candidate predictor variables were included in the multivariate logistic regression analysis. The results showed that age, drinking, ECLN, echogenicity, shape, calcification, TSH and K were the eight independent factors found to differentiate PTC from benign thyroid nodules (*P* < 0.05) (Table 3). These independent factors were used to construct a risk estimation nomogram for PTC (Fig. 2). According to the ruler above the corresponding nomogram of each risk factor, a single score of this factor is obtained. The total score can obtain the incidence of PTC for the corresponding patient. The higher the total score, the greater the possibility of the risk of PTC.

**Table 3:** Multivariate analysis of the relationship between PTC and benign nodules

	β	Odds ratio	95%CI	*P*
Age	-0.045	0.956	0.945–1.574	< 0.001
BMI	0.022	1.022	0.984–1.062	0.258
Location				
Isthmus	Ref	-	-	-
Upper pole	0.143	1.153	0.579–2.247	0.679
Middle third	-0.050	0.951	0.496–1.778	0.877
Lower pole	-0.174	0.84	0.437–1.574	0.593
Smoking	0.114	1.121	0.632–2.009	0.698
Drinking	0.629	1.862	1.013–3.469	0.047
Heart disease	-0.205	0.814	0.533–1.253	0.346
Enlarged cervical lymph nodes (ECLN)	0.461	1.586	1.116–2.283	0.012
K	-0.738	0.478	0.327–0.695	**< **0.001
TT3	-0.109	0.897	0.641–1.269	0.531
TSH	0.132	1.141	1.053–1.237	0.001
UA	0.001	1.001	0.999–1.002	0.176
HDL	0.123	1.131	0.715–1.795	0.598
RBC	-0.078	0.924	0.682–1.255	0.614
MCH	-0.111	0.895	0.785–1.021	0.096
RDW-SD	-0.045	0.956	0.912–1.001	0.0537
MCV	0.034	1.035	0.976–1.097	0.246
Echogenicity	3.369	29.07	14.572–66.880	**< **0.001
Boundary	0.176	1.193	0.860–1.642	0.286
Shape	1.923	6.838	4.945–9.534	**< **0.001
Intranodular Hyperechogenicity	0.137	1.147	0.836–1.578	0.395
CDFI	0.113	1.119	0.862–1.454	0.398
Calcification	0.459	1.583	1.124–2.233	0.008

### Predictive value of PTC occurrence in the training and validation groups

**Fig. 3:**
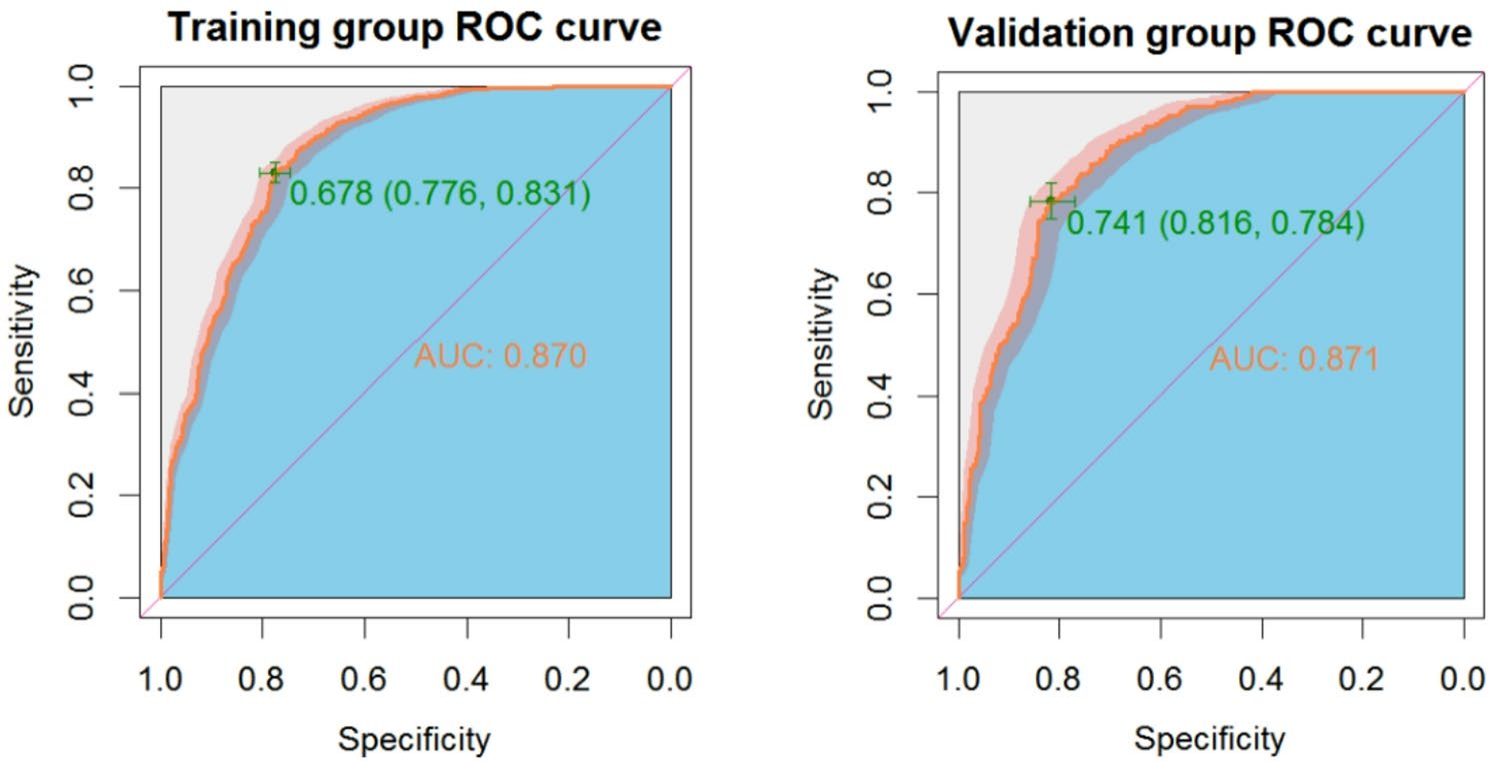
The ROC curves of the nomogram for PTC risk (left: training group; right: validation group). Notes: In the training group, the AUC were 0.869 (95% CI, 0.8527–0.8862); in the validation group, the AUC were 0.872 (95% CI, 0.8468–0.8970), respectively. ROC, receiver operating characteristics curve; AUC, area under curve

The ROC curve was drawn for the prediction effect of the nomogram (Fig. 3). The area under the ROC curve of the training group nomogram prediction model was 0.869 (95% CI, 0.853–0.886), the diagnostic cut-off point was 0.679, sensitivity 0.830 and specificity 0.776. When validated by using the validation group dataset, the area under the ROC curve of the predicted nomogram was 0.872 (95% CI, 0.847–0.897), the diagnostic cut-off point was 0.681, sensitivity 0.832 and specificity 0.768. In addition, the area under the curve (AUC) value of the validation group was only 0.012 lower than that of the training group, indicating that the prediction model has good prediction discrimination in both groups.

### Calibration curve of the nomogram

**Fig. 4:**
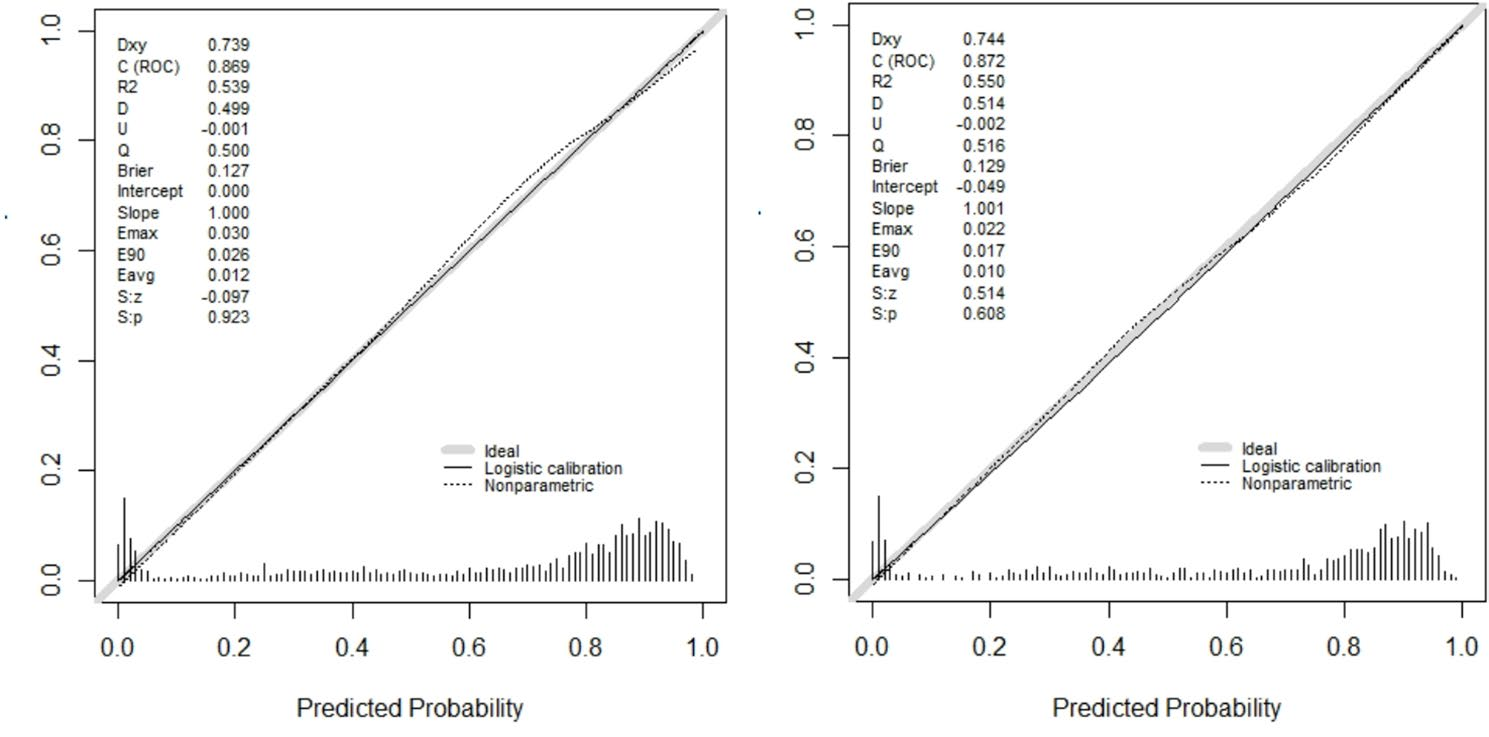
Calibration curve of the nomogram (left: training group; right: validation group). Notes: The diagonal dotted line represents a perfect prediction by an ideal model. The solid line represents the performance of the nomogram, of which a closer fit to the diagonal dotted line represents a better prediction

The calibration of the prediction model was evaluated by the Hosmer-Lemeshow goodness-of-fit test. In the training and validation groups, the calibration curve (Fig. 4) of the nomogram showed good agreement between the prediction and actual values; the Hosmer-Lemeshow goodness-of-fit test values were *P* = 0.923 and *P* = 0.608, respectively. This indicates that the model aligns well with the observed data

### Decision curve analysis (DCA) curve of the nomogram

**Fig. 5:**
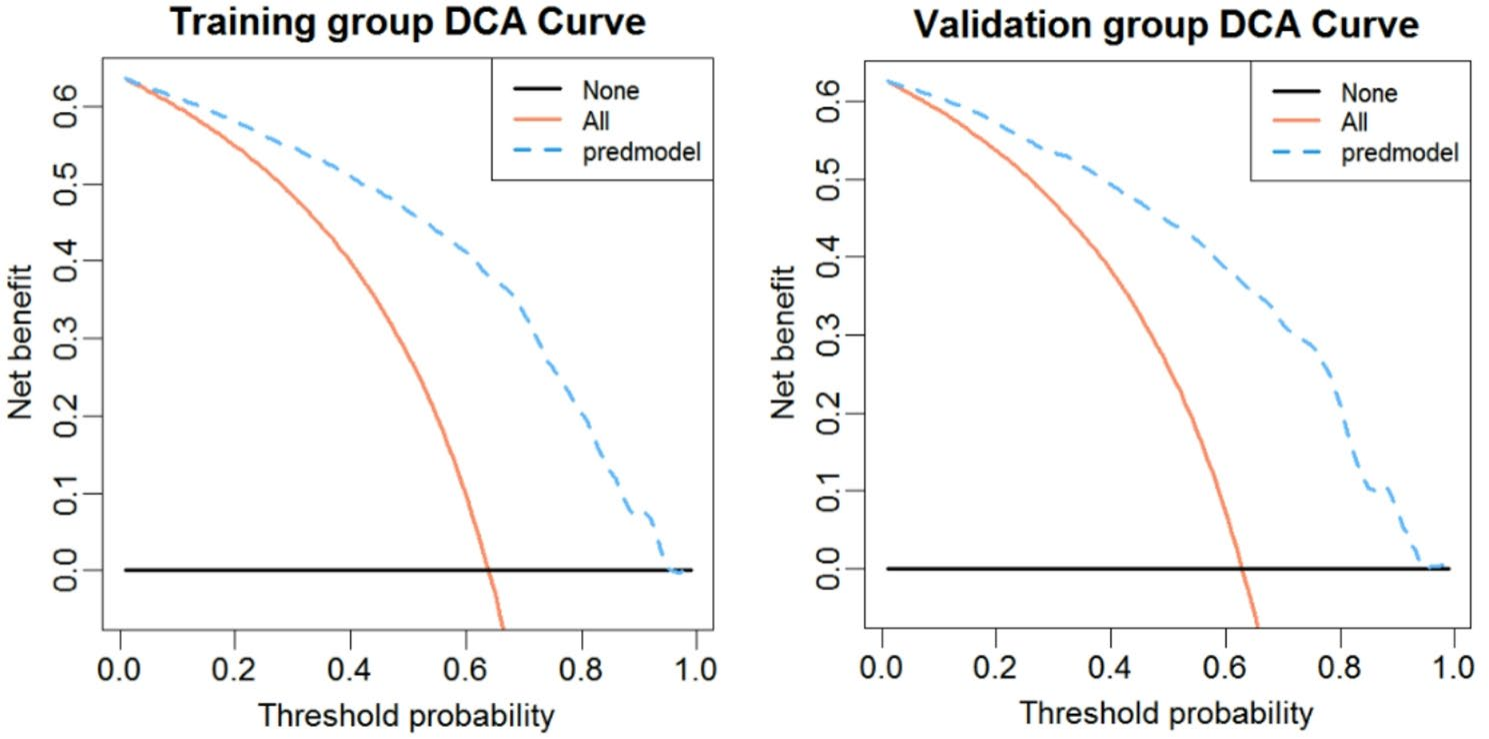
Decision curve analysis (DCA) of the nomogram (left: training group, right: validation group). Notes: The black solid line represents the net benefit when no participant is considered to exhibit PTC, while the brown solid line represents the net benefit when all participants are considered to suffer from PTC. The area between the model curve, “treat none line” (black solid line) and “treat all line” (brown solid line), represents the clinical usefulness of the model. The further the model curve is from the black and brown solid lines, the better clinical value the nomogram holds

Results of the DCA curve in the training and validation groups are shown in Fig. 5. The blue dashed line represents the model. The brown solid line represents the net benefit when all participants had PTC, while the black solid line represents the net benefit when no participant had PTC. The area between the “brown solid line” and “black solid line” of the model curve shows the clinical validity of the model. If the blue dashed line is above the horizontal black solid line and the left brown solid line, we can assume that the blue dashed line value of this section can be of benefit. The DCA curve shows that using the nomogram model to predict the occurrence of PTC risk has a higher net income. The nomogram model is a good assessment tool because of the available selectivity threshold probability.

### Novel risk stratification based on the predictive nomogram

**Fig. 6:**
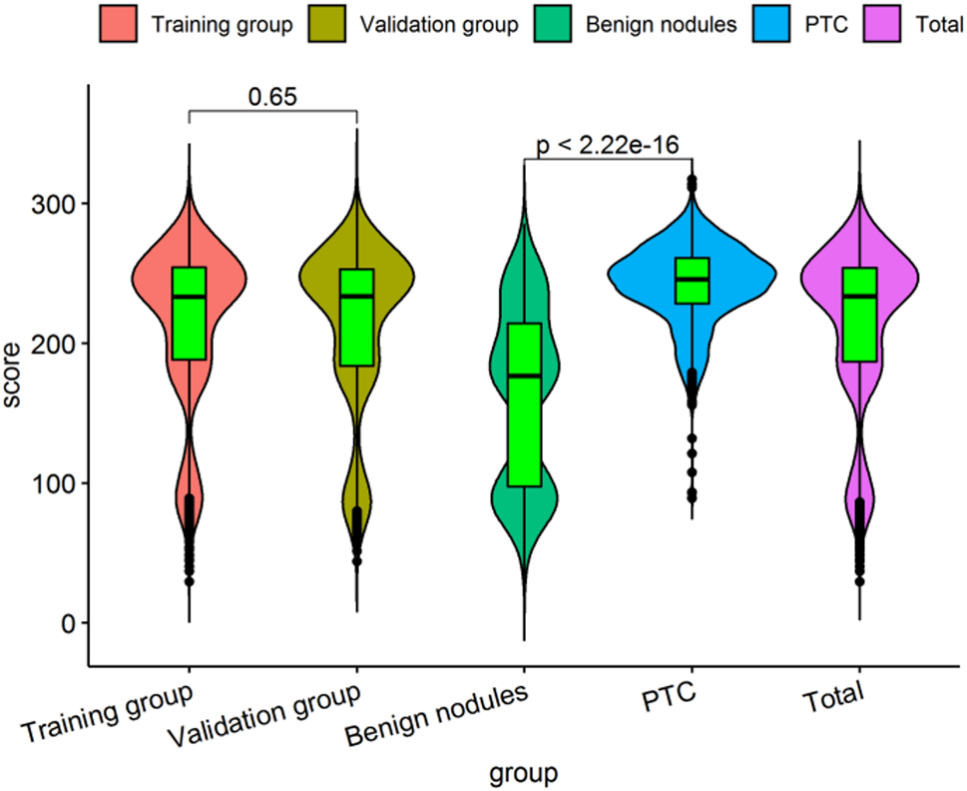
The fractional frequency distribution of PTCs and benign nodules groups estimated by the nomogram. Notes: The length of the violin plot indicates the range of scores, the width indicates the frequency of scores. The violin plot contains a green boxplot in the middle, and the black horizontal line in the middle of the boxplot indicates the average score

Each variable contained in the nomogram has its corresponding risk point, and the total risk score calculated for all patients can quantitatively predict their respective PTC risk. The fractional frequency distribution of each group estimated by the nomogram is shown in Fig. 6. As shown in Fig. 6, there was no significant difference in the scores of the training group and the validation group, *P* = 0.650. The scores of the group with PTCs were significantly higher than those of the group with (pls check this is correct) benign nodules (*P* < 0.001). There was a wave peak between 0 and 160 for the training and validation groups. This study sets this wave peak as extreme low-risk (ELR), mainly derived from the benign nodules group score. Another wave peak appears between the points 161 and 190 points, which was set as the low-risk (LR), also mainly derived from the benign nodules group score. The frequency number began to grow between 191 and 220 points, and was designated as moderate-risk (MR). At this point, the frequency of the PTC group gradually increases, while the benign nodule score gradually decreases. The maximum frequency was > 220, and high-risk (HR) was set here; this was mainly derived from the group with PTCs. We thereby determined three cut-off values (160, 190, 220) by using recursive partition analysis. As shown in Table 3, we established four subgroups as follows: (1) extreme low-risk group (patients with the nomogram score of ≤ 160), (2) low-risk group (160 < risk score ≤ 190), (3) moderate-risk group (190 < risk score ≤ 220), and (4) high-risk group (patients with a score of > 220). In the training group, the rates of PTC for extreme low-, low-, moderate-, and high-risk groups were 3.19%, 31.09%, 58.36%, and 86.65%, respectively (*P* < 0.001). Similarly, in the validation group, the rates of PTC for extreme low-, low-, moderate-, and high-risk groups were 2.16%, 36.04%, 53.64%, and 85.87%, respectively (*P* < 0.001). The above specific results are shown in Table 4. Further investigation was conducted to determine whether the relative risks for PTC across different risk categories identified by the nomogram were significantly different from one another. After paired comparison, we found there were significant differences between all groups.

**Table 4:** Risk stratification of patients with PTC based on risk scores of the nomogram model

Nomogram	ELR	LR	MR	HR	Total	*P* value	ELR-LR	ELR-MR	ELR-HR	LR-MR	LR-HR	MR-HR
	0-160	161–190	191–220	> 220			*P* value	*P* value	*P* value	*P* value	*P* value	*P* value
Training data set												
PTC	10 (3.19)	74 (31.09)	171(58.36)	1084(86.65)	1339	< 0.001	< 0.001	< 0.001	< 0.001	< 0.001	< 0.001	< 0.001
Nodular goiter	303 (96.81)	164(68.91)	122 (41.64)	167 (13.34)	756							
Total	313	238	293	1251	2095							
Validation data set												
PTC	3 (2.16)	40 (36.04)	59 (53.64)	462 (85.87)	564	< 0.001	0.018	< 0.001	< 0.001	0.009	< 0.001	< 0.001
Nodular goiter	136 (97.84)	71 (63.96)	51 (46.36)	76(14.13)	334							
Total	139	111	110	538	898							

### Probability density curves of some indices

**Fig. 7:**
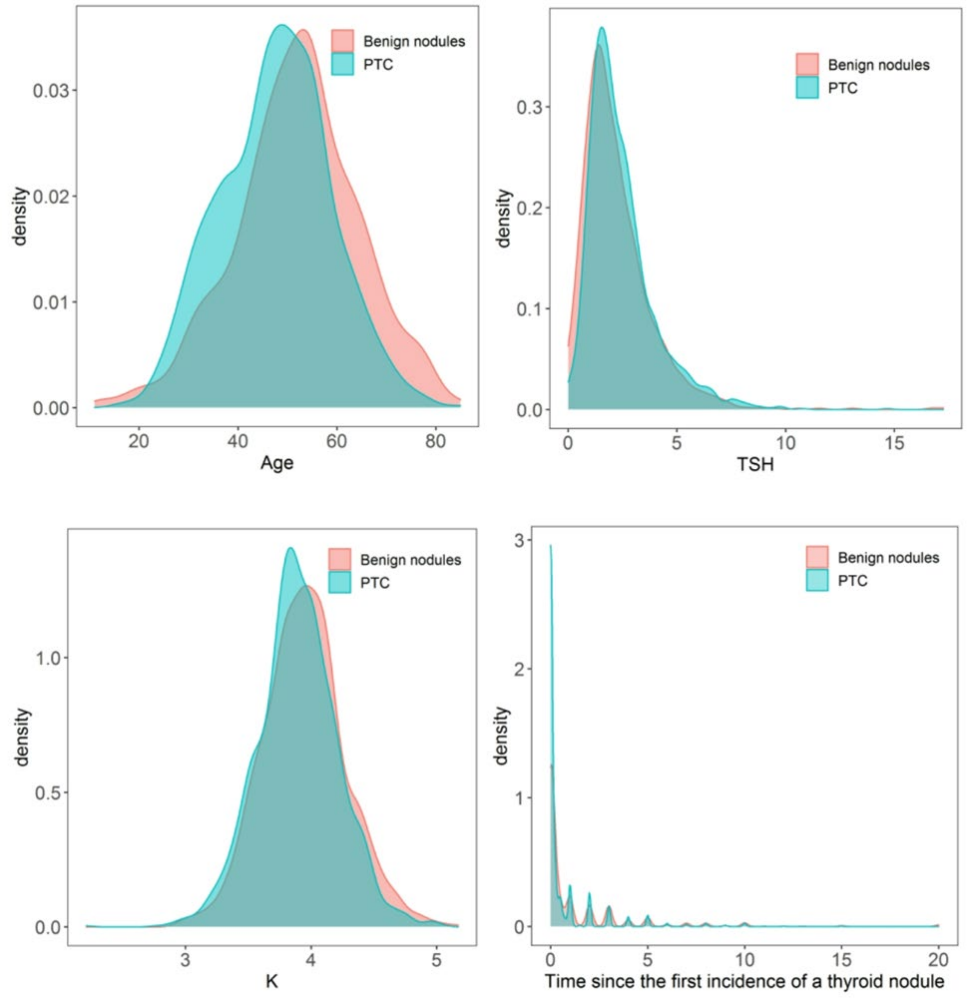
Probability density curves of PTC and benign nodules in age, TSH, K and time since the first incidence of a thyroid nodule. Note: The X-axis represents the variable, and the Y-axis represents density. The higher the value of density, the higher the proportion

We present the probability density curves of some indicators in the PTC and benign nodule groups, containing the data of all patients in the training and validation groups (Fig. 7). The average age is (47.64 ± 11.16 ) years of age in the PTC group, and (52.01 ± 12.51) years of age in the benign nodules group. The average TSH level is (2.45 ± 1.54uIU/ml) in the PTC group, and (2.25 ± 1.65uIU/ml) in the benign nodules group. The average K level is (3.89 ± 0.32mmol/L) in the PTC group, and (3.95 ± 0.33mmol/L) in the benign nodules group. The time since the first incidence of a thyroid nodule is (0.65 ± 1.55years) in the PTC group, and (1.28 ± 2.67years) in the benign nodules group.

## Discussion

At present, some guidelines and consensus have limited value for assessing the individual risk of malignant thyroid nodules because they do not take certain clinical factors (such as age, gender, and family history of thyroid cancer), or biochemical factors (such as TSH levels or the presence of autoimmune thyroiditis) into account. Certain individual characteristics are highly associated with thyroid malignancy, but no single feature can be considered foolproof. For these reasons, several authors have developed different predictive models to assess the risk of thyroid nodule malignancy that combine clinical, analytical, and ultrasound variables [14–22]. These can help clinicians and patients improve clinical decision-making. Nomograms are statistical tools that are ideally suited to individualizing risk assessment and have been used for a variety of situations [23]. In this study, we integrated the clinical characteristics, biochemical profiles, and ultrasonographic and cytologic features into a nomogram and predicted the risk of malignancy in thyroid nodules. This nomogram, which improves feasibility and practical appeal, helps clinicians with decision-making and reduces unnecessary invasions.

This study is retrospective research based on patients undergoing surgery for thyroid nodules at The Hospital of Traditional Chinese Medicine Affiliated to Xinjiang Medical University, aiming to predict the risk of PTC among these patients. This study suggests that, age, drinking, ECLN, K, TSH, echogenicity, shape and calcification are independent risk factors for PTC. Drinking, ECLN, TSH, echogenicity (hypoechogenicity), shape (irregular shape), and calcification positively correlate with PTC. Age and K correlate negatively with PTC. Among the above indicators, echogenicity, shape, age, drinking, ECLN and calcification are the most strongly correlated indicators. This therefore shows that hypoechogenicity, irregular shape, drinking, ECLN and calcification are the high-risk factors of PTC. Original data was randomly divided into two groups: a training data set (*n* = 2095) and a validation data set (*n* = 898). The verification results show both have good risk prediction ability. The calibration chart shows that the nomogram is accurate in predicting the risk of PTC. Meanwhile, our decision curve analysis also confirms the clinical utility value of the nomogram.

Among the independent risk factors in this PTC prediction model, hypoechogenicity is the factor with the greatest risk weight (OR = 29.07, 95%CI, 14.572–66.880, *P* < 0.001). Hypoechogenicity is an important feature of thyroid color ultrasound in the description of thyroid nodules. Ultrasound is the low-cost, noninvasive, rapid and reproducible method for the examination, diagnosis and follow-up of thyroid nodules [24]. Nodules with a vertical orientation, an ill-defined or irregular margin (including extrathyroidal extension), microcalcifications that were solid, and markedly hypoechoic, were positively associated with malignancy, while comet-tail artifacts were negatively associated with malignancy [25]. The study by Chen et al. focused on the features of the ultrasound images and carefully analyzed these characteristics to construct a modified risk stratification system tailored for the Chinese population. It is important to clarify that the Chinese TI-RADS is not a modified version of the ACR TI-RADS, it is another version of TI-RADS [26]. This model showed high sensitivity and specificity for predicting thyroid cancer in our selected series of patients with thyroid nodules. The study of Arpanay et al. [27] indicates that hypoechogenicity, ambiguous margins, and microcalcification are independent predictors of malignancy, and can be independently used as screening tools to identify a high risk of malignancy. European Thyroid Association (ETA) guidelines suggest that in the case of nodules with heterogeneous echogenicity of the solid component, the presence of any hypoechoic tissue should classify the nodule as intermediate risk [28]. In a study by Lee et al. [29], in which stratification of the malignant risk of thyroid nodules was based on the degree of hypoechogenicity, the final findings also suggested that heterogeneous predominantly hypoechoic thyroid nodules showed a significantly higher malignancy risk than predominantly iso- or hyperechoic thyroid nodules (*p* < 0.001). In this study, although the degree of hypoechogenicity was not further subclassified, this did not compromise the identification of final risk factors, and results consistent with those from studies in diverse regions worldwide were obtained. While the present study focuses on papillary thyroid carcinoma, relevant studies on other types of thyroid cancer were also reviewed. There are studies suggesting that follicular variant PTCs and follicular carcinomas more frequently show iso- or hyperechogenicity than conventional PTCs [30, 31]. It also demonstrates that the population included in our study was predominated by conventional PTCs. Attention to this point is therefore required when utilizing this nomogram for clinical predictions.

Our study suggests that irregular shape (OR = 6.838, 95%CI, 4.945–9.534, *P* < 0.001), calcification (OR = 1.583, 95%CI, 1.124–2.233, *P* = 0.008), ECLN (OR = 1.586, 95%CI, 1.116–2.283, *P* = 0.012), and drinking (OR = 1.862, 95%CI, 1.013–3.469, *P* = 0.047) are also high-risk factors for the occurrence of PTC. It has been pointed out in the previous paragraph that irregular shape and calcification of nodules are all ultrasound malignant manifestations of thyroid nodules. Zhao and Raposo [32, 33] have also shown that irregular shape and calcification are independent risk factors for thyroid cancer, although calcification is commonly seen in thyroid ultrasound images of both benign and malignant nodules. However, most studies suggest that the incidence of malignant tumors in patients with calcification is higher than that of benign tumors in patients with calcification, therefore, calcification can be used to predict thyroid cancer [34]. Calcification of malignant thyroid lesions usually results from the proliferation of blood vessels and dense fibrous tissue and the deposition of calcium salts [35]. According to the calcification pattern, microcalcification and intranodular calcification were significantly associated with malignancy [36]. Microcalcification is considered the most specific ultrasound indicator in the diagnosis of papillary thyroid carcinoma [34]. However, other patterns of calcification are of unclear clinical significance [37]. In the present study, calcification types were not subclassified, and consistent verification of this parameter is intended in future investigations. Cervical lymph nodes are a common area for metastasis of malignant thyroid cells through lymphatic drainage [38, 39]. Mohamed et al. [40] found that the risk of thyroid nodule malignancy increased with the presence of suspicious ultrasound features on cervical lymph nodes. Research from 2010 by Hands et al. [41] showed a correlation between the presence of benign ECLN, found on preoperative neck USG, and the risk of PTC in thyroid nodules. Meanwhile, the findings of Mohamed et al. [40] show that the presence of ECLN increases predictive value in diagnosing PTC and in suspicious thyroid nodules. The presence of an ECLN on the preoperative neck USG can provide valuable information to help the surgeon determine the optimal surgical treatment for patients with suspected thyroid nodules [41]. This study also confirms that ECLN is an independent risk factor for papillary thyroid cancer. We believe the above studies will encourage further research to investigate the association between ECLN and papillary thyroid cancer. Many meta-analyses have been conducted on the relationship between alcohol consumption and cancer, and the following conclusions have been drawn [42–45]. The study by Choi et al. [43] has proposed that very light drinking or light drinking was not associated with the incidence of most cancers, except for female breast cancer in women and male colorectal cancer. Conversely, light drinking was associated with a decreased incidence of both female and male thyroid cancer, with marginal significance. The study by De Menezes et al. [44] also showed that alcohol consumption may be inversely associated with thyroid cancer. These conclusions are inconsistent with those of our study; the reason for this may also depend on the dose of alcohol and the population studied. We only performed a qualitative analysis of alcohol consumption, but not a quantitative. In subsequent studies, we will make further quantitative analysis on the effects of alcohol consumption on thyroid cancer to provide more clinical evidence for the clinic.

This study suggests that younger age (OR = 0.956, 95%CI, 0.945–1.574, *P* < 0.001), TSH (OR = 1.141, 95%CI, 1.053–1.237, *P* = 0.001) and K (OR = 0.478, 95%CI, 0.327–0.695, *P* < 0.001) are independent risk factors for PTC. Age is a well-established prognostic factor for thyroid cancer survival [46], and it is included in the American Joint Committee on Cancer (AJCC) thyroid cancer-staging system [47]. Our study demonstrates an inverse association between age and papillary thyroid carcinoma. Previous studies have also provided us with some clues on the association of age and thyroid cancer. Relative to normal thyroid tissue, thyroid cancer is characterized by reduced sodium iodine symporter expression [48]. Studies have shown that patient age is associated with variations in the expression of the sodium-iodine symporter, which plays a crucial role in radioiodine uptake [49–51]. It is not entirely clear why young patients are more likely to develop radioiodine-dependent disease. There is controversy, but it is possible that specific genetic mutations, such as the somatic rearrangement forms of RET (RET/PEC rearrangement), have a higher incidence in youths, while BRAF mutations are more common in adults [52–54]. As for the impact of potassium (K) in thyroid disease, there are no relevant reports in the literature, and we also hold an exploratory attitude towards our conclusions, which need more theoretical and data support. Increasing evidence suggests that the serum concentration of TSH is an independent predictor for the diagnosis of thyroid malignancy in patients with nodular thyroid disease [55–57]. Moreover, there are higher TSH concentrations in patients with more malignant preoperative serum, suggesting that TSH may play a role in the progression of differentiated thyroid cancer. The lower cancer incidence may be explained by the fact that the low malignant potential of TSHR-mutant thyroid nodules is attributed to the predominance of Gαs-cAMP signaling and the extremely rare activation of the Gβγ-mediated Ras-MAPK/PI3Kγ oncogenic pathway, which aligns with the low cancer incidence observed in these nodules [58]. Taken together, these findings suggest that serum TSH concentrations can be used as a diagnostic adjunct in the identification of high-risk patients, who require further investigation and/or surgical intervention [59]. Our study confirms that an elevated level of TSH is an independent risk factor of PTC, even if it remains in the normal range. Our study is retrospective and the sample of patients is probably too small to make conclusions. Further studies are needed to evaluate the role of TSH in the initiation and progression of thyroid cancer.

## Limitations

This study, based on clinical, biochemical, and ultrasound features of the nomogram, has a good degree of differentiation. At the same time, the calibration plot and DCA results of the line graph prove that the prediction model has good discrimination. This confirms the clinical application value of the model. The present study still has the following limitations: (1) The external validation of the nomogram in this study came from the same hospital, which may present a case selection bias. Further analysis and evaluation should therefore be carried out in combination with multi-center large-sample clinical data. (2) A larger sample size of clinical data is still needed to improve the validity and reliability of the model. (3) Some data in this study needs further stratified and quantitative research. (4) This study is retrospective, and prospective studies in a larger patient population are required to define and verify this model of risk prediction to improve clinical management. (5) The assessment of enlarged cervical lymph nodes (ECLN) in this study was based on routine ultrasound reports. While the presence of ECLN was a significant predictor, a more detailed analysis of specific malignant features, such as cystic changes, calcifications, and internal echogenicity, was not systematically performed. The inclusion of these features in future prospective studies could enhance the predictive value of lymph node status. (6) Although hypoechogenicity was identified as a strong independent predictor of malignancy in our model (OR = 29.07), our study did not stratify hypoechoic nodules by degree of echogenicity (e.g., mild, moderate, or marked). Previous studies, including a recent application within the Chinese TI-RADS framework [60], have demonstrated that the malignancy risk increases with the degree of hypoechogenicity, with mildly hypoechoic nodules exhibiting a significantly lower risk compared to moderately or markedly hypoechoic ones. In particular, recent evidence suggests that mildly hypoechoic solid nodules (e.g., TI-RADS 4) may not show a significant association with malignancy, whereas more pronounced hypoechogenicity carries a proportionally higher risk [61]. The inclusion of such a stratification in future studies could potentially improve the discriminatory accuracy of the nomogram and allow for more granular risk assessment.

## Conclusions

Among thyroid malignancies, papillary thyroid carcinoma has the highest incidence. It is therefore crucial to identify PTC and benign thyroid nodules to avoid unnecessary overtreatment, such as fine needle aspiration (FNA) biopsy and surgery, and to search for the risk factors for PTC. In this study, we propose a nomogram model combining clinical, biochemical, and ultrasound features. The visualization of the nomogram model can provide reference for clinical workers for screening and early diagnosis of PTC by including patient information in the risk prediction model and calculating the risk score to assess the risk of PTC in patients. In this study, a nomogram model integrating clinical, biochemical, and ultrasound features is proposed. Through incorporating patient-specific information into the risk prediction framework, calculating corresponding risk scores, and visualizing the model, it provides a valuable reference for clinicians in the screening and early diagnosis of papillary thyroid carcinoma (PTC).

## Data Availability

The original contributions presented in the study are included in the article/Supplementary Material. Further inquiries can be directed to the corresponding authors.

## Abbreviations

PTC: Papillary thyroid carcinoma
ECLN: Enlarged cervical lymph nodes
TSH: Higher thyroid stimulating hormone
FNA: Fine needle aspiration
TI-RADS: Thyroid Imaging Reporting and Data System
BMI: Body mass index
ATA: American Thyroid Association
TG: Triglyceride
CHOL: Cholesterol
HDL: High-density lipoprotein
LDL: Low-density lipoprotein
T3: Triiodothyronine
T4: Thyroxine
FT3: Free triiodothyronine
FT4: Free thyroxine
TSH: Thyroid-stimulating hormone
TRAb: Thyrotropin receptor antibody
TGAb: Antithyroglobulin antibodies
TPOAb: Thyroid peroxidase antibodies
Tg: Thyroglobulin
AFP: Alpha-fetoprotein
CEA: Carcinoembryonic antigen
CT: Calcitonin
CDFI: Color Doppler flow imaging
AJCC: American Joint Committee on Cancer
ROC: Receiver operating characteristic
AUC: Area under the curve
DCA: Decision curve analysis
LR: Low-risk
MR: Moderate-risk
HR: High-risk
CI: Confidence interval
OR: Odds ratio
